# Controlled-Release from High-Loaded Reservoir-Type Systems—A Case Study of Ethylene-Vinyl Acetate and Progesterone

**DOI:** 10.3390/pharmaceutics12020103

**Published:** 2020-01-28

**Authors:** Ioannis Koutsamanis, Amrit Paudel, Klaus Nickisch, Karin Eggenreich, Eva Roblegg, Simone Eder

**Affiliations:** 1 Research Center Pharmaceutical Engineering GmbH, Inffeldgasse 13, 8010 Graz, Austria; ioannis.koutsamanis@rcpe.at (I.K.); amrit.paudel@rcpe.at (A.P.); 2 Institute of Pharmaceutical Sciences, Department Pharmaceutical Technology and Biopharmacy, University of Graz, Universitaetsplatz 1, 8010 Graz, Austria; 3 Institute of Process and Particle Engineering, Graz University of Technology, Inffeldgasse 13, 8010 Graz, Austria; 4 Evestra Inc., 6410 Tri County Parkway, Schertz, TX 78154, USA; knickisch@evestra.com (K.N.); KEggenreich@evestra.com (K.E.)

**Keywords:** vaginal drug delivery, controlled release, thermoplastic polymer, hot-melt extrusion, drug diffusivity

## Abstract

Reservoir systems (drug-loaded core surrounded by drug-free membrane) provide long-term controlled drug release. This is especially beneficial for drug delivery to specific body regions including the vagina. In this study, we investigated the potential of reservoir systems to provide high drug release rates over several weeks. The considered model system was an intra-vaginal ring (IVR) delivering progesterone (P4) in the mg/day range using ethylene-vinyl acetate (EVA) as release rate-controlling polymers. To circumvent the high material needs associated with IVR manufacturing, we implemented a small-scale screening procedure that predicts the drug release from IVRs. Formulations were designed based on the solubility and diffusivity of P4 in EVAs with varying vinyl acetate content. High in-vitro P4 release was achieved by (i) high P4 solubility in the core polymer; (ii) high P4 partition coefficient between the membrane and the core; and/or (iii) low membrane thicknesses. It was challenging for systems designed to release comparatively high fractions of P4 at early times to retain a constant drug release over a long time. P4 crystal dissolution in the core could not counterbalance drug diffusion through the membrane and drug crystal dissolution was found to be the rate-limiting step. Overall, high P4 release rates can be achieved from EVA-based reservoir systems

## 1. Introduction

Controlled drug delivery systems (CDDSs) are advantageous over conventional dosage forms as they yield more stable plasma profiles, allow for reduced administration frequency, and minimize potential of side-effects [1]. While conventional dosage forms release the drug within a comparatively short time frame, CDDSs contain polymers to control drug release and thereby, the onset and duration of the drug’s action in the body. Polymer-based CDDSs are classified into three types: (i) polymer-drug conjugates, (ii) monolithic matrix systems and (iii) reservoir-type systems [2]. In conjugates, drugs are covalently bound to a water-soluble/biodegradable polymer. In matrix systems, active pharmaceutical ingredients (APIs) are homogeneously dispersed/dissolved in the release controlling polymer. In reservoir systems, the drug is embedded in a polymeric core, which is similar to matrix systems, but the core is surrounded by a drug-free polymer membrane. Thereby, the membrane’s thickness and the drug´s permeability through the polymer control the drug release [3]. More precisely, ideal reservoir systems contain the dissolved drug in the core polymer together with drug crystals that act as a reservoir [4]. Once dissolved drug molecules diffuse through the membrane, drug crystals dissolve in the core polymer and a constant drug concentration gradient across the membrane is provided. Thereby, constant drug release is achieved over several weeks [5,6,7], months [8,9], to even years [10,11]. This makes reservoir systems most desirable for mid- and long-term administration to specific body regions such as the vagina, uterus or eye. In addition, the membrane may prevent recrystallization of the active candidate on the surface of the respective device.

One of the first release-regulating polymers that is still used in subdermal and ophthalmic implants, intrauterine devices (IUDs) and intra-vaginal rings (IVRs) is ethylene-vinyl acetate (EVA) [12]. It is a thermoplastic, random block copolymer of ethylene and vinyl acetate (VA) units that controls drug release via diffusion. The VA content ranges between 5 and 40% and determines the polymer´s amorphous content and consequently, the drug´s diffusivity [13,14]. Currently, there are two marketed EVA-based reservoir-type products available for long term hormonal delivery, namely Nexplanon^®^, a transdermal implant, and NuvaRing^®^ (with its generic products), an IVR. Both are used for contraceptive purposes and release rather low amounts of hormones (in the µg/day range) over 3 weeks (NuvaRing^®^) and 3 years (Nexplanon^®^). To the best of the authors´ knowledge, studies addressing reservoir systems that are designed to provide high and constant drug release (in the mg/day range) are scarce. Thus, the present study aimed at elucidating the potential and limitations of high-loaded, high-releasing model reservoir systems using EVA with different VA contents as release regulating polymers. Progesterone (P4), which is currently available in a silicon-based matrix IVR (Progering^®^), served as model drug. When delivered intra-vaginally, P4 doses are 5 and 10 mg/day for hormonal contraception and hormone replacement therapy respectively [15,16]. Therefore, in our study, we defined both target release rates. In an attempt to save material and time, we applied and further developed small-scale systems that were recently shown to predict the performance of IVRs by our group [17,18]. In a first step, we rationally designed reservoir systems based on the physicochemical properties of the selected drug and the polymers. In a second step, we manufactured and thoroughly characterized the reservoir systems. Finally, we elucidated critical factors affecting the in vitro release of P4 from the designed systems.

## 2. Materials and Methods 

### 2.1. Materials

Micronized P4 was supplied by Euro OTC Pharma (particle size: x95 10 µm, Bönen, Germany). The EVA polymers used were EVA9 (Greenflex ML31, 9% VA, Versalis, Milan, Italy), EVA18 (ATEVA 1820, 18% VA, Celanese, Irving, TX, USA), EVA28 (Greenflex ML60, 28% VA, Versalis, Milan, Italy) and EVA40 (ELVAX 40L_03, 40% VA, DuPont de Nemours, GmbH, Neu-Isenburg, Germany). For the preparation of the dissolution medium, Milli-Q water (in-house preparation, MicroPure TKA), glacial acetic acid (Merck KGaA, Darmstadt, Germany), sodium lauryl sulfate (SLS) and sodium acetate trihydrate (both Carl Roth GmbH + Co. KG, Karlsruhe, Germany) were used. For the mobile phase, ethanol of HPLC grade (Sigma Aldrich, Vienna, Austria) and MilliQ water were applied. 

### 2.2. Methods

#### 2.2.1. Thermal Analysis of the Pure Components

The polymers and P4 were analyzed via modulated differential scanning calorimetry (mDSC). A 204 F1 Phoenix apparatus (Netzsch, Selb, Germany) equipped with an external cooling system and an automated sampling unit was used. Samples (5–10 mg each) were placed into aluminum pans, which were subsequently closed with pierced lids. Samples were heated from −60 to 150 °C with a heating rate of 5 °C/min (0.53 °C amplitude, 40 s period), kept at this temperature for 5 min and thereafter, cooled to −60 °C with a cooling rate of −10 °C/min. The heating and cooling cycle were repeated. Nitrogen was used as the purge gas (flow rate of 50 mL/min). The obtained thermograms were analyzed with the Netzsch Proteus analysis software. The melting temperature (onset) of crystalline components and corresponding enthalpy of fusion were determined from the total heat flow signal. The glass transition temperature (T_g_) was determined from the reversing heat flow signal. The crystallinity of the EVA polymers was calculated by Xcr (%) = ΔH/ΔH0, where ΔH is the enthalpy of fusion associated with the melting of the crystalline components of EVA and ΔH0 is the enthalpy of fusion of the pure polyethylene crystal (292.3 J/g) [19].

#### 2.2.2. P4 Solubility in EVA Polymers

The equilibrium solubility of P4 in EVA polymers at 37 °C was determined using thin EVA films incubated in an aqueous suspension comprising an excess of P4. The equilibrium solubility was defined as the concentration of P4 quantified in the EVA film once the partitioning equilibrium of P4 between the aqueous and polymer phase had been reached [20]. The polymer films (250 µm thickness) were produced via vacuum compression molding (VCM, MeltPrep, GmbH, Graz, Austria) following a procedure previously described [18]. The films were cut into squares of 2 × 2 cm and placed into glass vials comprising 15 mL of deionized water and 10 mg of P4 (P4 solubility in water at 37 °C: 11.9 ± 1.0 µg/mL). The vials were placed into an incubator shaker operated at 130 rpm and 37 ± 0.5 °C. Films were withdrawn after 3, 4 and 5 weeks and carefully rinsed with deionized water to remove excess drug from the surface. P4 was extracted from the films with 50 mL ethanol in an incubator shaker operated at 40 °C and 300 rpm over 2 h and quantified in the supernatant using ultra-performance liquid chromatography (UPLC; details are provided in Section 2.2.9). Each polymer and time point were investigated in triplicate.

Since EVA-based systems are manufactured via thermal processing, the effect of thermal treatment on the P4 solubility in potential core polymers (i.e., EVA28 and EVA40) was evaluated. To this end, a method reported by our group was used [18]. Briefly, EVA28 was loaded with 4, 5 and 6 wt.% and EVA40 with 5, 6 and 7 wt.% P4 via impregnation using tetrahydrofuran (THF). The drug-loaded polymers were processed via the VCM tool at 110 °C (i.e., thermal processing) and the obtained VCM samples were stored in sealed LDPE bags at 37 °C for 14 days to allow for potential precipitation of excess drug via phase separation and/or crystallization. Thereafter, the samples were analyzed via hot-stage polarized light microscopy (HS-PLM; Olympus BX51M microscope coupled with a temperature-controlled stage LINKAM Scientific Instruments, THMS 600/720, Tadworth, UK). The samples were heated to 90 °C with a rapid heating rate of 80 °C/min. This temperature is above the melting of EVA28 and EVA40 (for details the reader is referred to Section 3.1) but below the melting of P4 (i.e., 104 °C for the lowest melting form III [21]). Therefore, any crystals present at this temperature can be assigned to crystalline P4. The fast heating rate impeded the dissolution of P4 crystals in the EVA melt during heating. The highest drug concentration, at which crystalline P4 was not detected, was nominally considered the P4 solubility after thermal treatment.

#### 2.2.3. P4 Diffusivity and Permeability in EVA Polymers

The diffusion coefficient of P4 in EVA polymers was determined following a method by Siepmann et al. [4]. This includes determination of the drug release from undersaturated polymer films and calculation of the diffusivity by treating the generated data. EVA polymers were loaded with 0.4 wt.% P4 via impregnation with THF, milled in a cryogenic mill (Retsch Cryomill, Haan, Germany) and processed into films of 250 µm thickness via the VCM tool. 50 mL acetate buffer (pH 4.5) supplemented with 1% SLS served as release medium, which provided sink conditions. The experiments were performed in an orbital incubator shaker at 37 ± 0.5 °C and 130 rpm. Samples of 1 mL were withdrawn after 30 min, 1, 2, 3, 4, 6, 8, 10 and 24 h and replaced by 1 mL fresh pre-heated medium. P4 was quantified in the samples via UPLC (Section 2.2.8). The cumulative fraction of P4 released was plotted against time and equation 1 was used to calculate the diffusion coefficient of P4 [4,22].

(1) $$M_{t}/M_{\infty }=1-(8/\pi^{2})\;\sum_{n=0}^{\infty }[1/(2n+1{)}^{2}]\;\exp[(-D(2n+1{)}^{2}\;\pi^{2}\;t)/L^{2}]$$

M_t_ is the cumulative drug release at time t (kg), M_∞_ is the drug release at infinite times (kg, equal to the drug loading), n is a summation index, D is the apparent diffusion coefficient of the drug within the polymer (m^2^/s) and L is the thickness of the film (m). Each polymer was investigated in triplicate. The permeability of P4 in the EVA polymers was calculated as the product of diffusivity and equilibrium solubility [23].

#### 2.2.4. Rational Formulation Design

The drug release from perfect reservoir systems of slab geometry is described by Equation (2).

Mt/t = (ADKC_s_)/L (2)

A is the diffusional surface area of the device (m^2^), K is the partition coefficient of the drug between the membrane and the core polymer (K = C_s_, membrane/C_s_, core) and C_s_ is the drug concentration gradient across the membrane (kg/m^3^), which is assumed as the solubility of the drug in the core polymer. 

Based on Equation (2) and the results of the solubility and diffusion coefficient of drug in the selected polymers (Section 3.2 and Section 3.3), reservoir systems were designed to provide specific daily P4 release rates. For IVRs, the required P4 target release rate is 5 and 10 mg/day [15,16]. In this study, small-scale VCM systems were used, which predict the drug release from the corresponding IVRs [17,18]. The VCM systems consist of a cylindrical core of 10 mm diameter and two EVA membranes attached to the flat circular surfaces of the core [17]. Considering the different diffusional areas of a VCM system (i.e., 1.57 cm^2^) and an IVR of NuvaRing^®^ dimensions (i.e., 19.35 cm^2^), the target P4 release from the VCM systems is 400 and 800 µg/day to achieve the 5 and 10 mg/day release from the corresponding IVRs.

For Cs both, the equilibrium solubility and solubility after thermal treatment were applied. Importantly, the drug loading needs to be chosen in the way that the initial drug concentration in the core polymer exceeds the drug solubility. Thereby, sufficient drug crystals that act as reservoir and allow for constant drug release are provided. We selected the initial drug concentration to be 9 times the equilibrium solubility.

During formulation design, different core/membrane polymer combinations and different membrane thicknesses were considered. In a first step, the core/membrane polymer combination and the membrane thickness were chosen to be similar to the NuvaRing^®^ formulation (i.e., EVA28 in the core and EVA9 in the membrane; membrane thickness 100 µm), as this yields suitable mechanical characteristics for an IVR [24]. In a second step, the VA content of the membrane polymer and/or the membrane thickness were adjusted to modify the P4 release rates. Finally, the core polymer was changed to EVA40. Again, different membrane EVA types and/or thickness were selected to yield the target release. In every case, the VA content of the membrane polymer was lower than the VA content of the core polymer. Thereby, the P4 permeability was lower in the membrane, making the P4 permeability in the membrane the rate-determining step during drug release. The membrane thicknesses ranged between 100 and 300 µm, since these thicknesses can be produced via hot melt co-extrusion [25] used for later IVR manufacturing

#### 2.2.5. Preparation of the VCM Systems

The core polymers, i.e., EVA28 and EVA40, were loaded with P4 via hot melt extrusion (HME). Binary blends (30 g) of EVA28 and 16 wt.% P4 (referred to as V1) and EVA40 and 32 wt.% P4 (referred to as V2) were prepared in a tumble blender (TURBULA^®^ T2F, Willy A. Bachofen AG, Muttenz, Switzerland) operated at 70 rpm for 40 min. The blends were manually fed into a table top extruder (ZE 9 HMI, Three-Tec GmbH, Seon, Switzerland) operated at a screw speed of 70 rpm and 110 °C. The extruder was equipped with two co-rotating kneading screws (9 mm diameter) and a cylindrical die of 1.5 mm. The extrudates were cooled at ambient conditions and stored at 5 °C prior to further characterization and processing into the VCM cores.

The core and the membranes were produced separately via the VCM technology and finally combined, to form the VCM systems of 10 mm diameter and 4 mm height Formulation details are given in Table 1. Details of the preparation method can be found elsewhere [17]. To prepare the membranes, 6.7–21 mg of EVA9, EVA18 or EVA28 - depending on the target membrane thickness - were processed at 140 °C (EVA9) and 110 °C (EVA18, EVA28). For the core preparation, 280 mg of V1 or V2 were processed at 110 °C. Finally, the core and the membranes were combined in the VCM tool at 110 °C. The VCM systems were investigated via 3D optical coherence tomography (OCT) to evaluate the integrity of the core/membrane interface and the membrane thickness (resolution: 4.1 µm axial, 10 µm lateral in focus). Image acquisition and evaluation were performed on 30 random points along the membrane surface via the vendor software (OSeeT 3.3, Phyllon, Austria) and ImageJ image processing software [26]. To equilibrate the drug distribution between the core and membrane, the VCM systems were stored at 37 °C over 14 days prior to further characterization.

#### 2.2.6. Solid State Characterization of the VCM Systems

The solid-state profile of P4 in the VCM core was investigated using HS-PLM, DSC and attenuated total reflectance Fourier transform infrared spectroscopy (ATR-FTIR). Samples were manually cut from the VCM systems (axial cuts) with a surgical blade. For every core formulation, one VCM system was investigated (i.e., V1_18_200 and V2_28_200, Table 1), as it can be assumed that the membrane does not influence the solid state of P4 in the core polymer. HS-PLM and DSC investigations were performed using the aforementioned equipment (see Section 3.1 and Section 3.2). For HS-PLM and DSC, the same temperature range and heating rate were applied, i.e., 25–160 °C and 5 °C/min. During HS-PLM, images were recorded every 1 °C with a magnification of 4×. For the ATR-FTIR measurements, an FTIR Vertex 70 (Bruker, Rheinstetten, Germany) equipped with a DLaTGS detector and an ATR unit (MVP Pro Star, Diamond crystal) was applied. Spectra were recorded at wavelengths ranging between 2000 and 600 cm^−1^ with a resolution of 2 cm^−1^. Every measurement was performed in triplicate.

#### 2.2.7. In-Vitro Drug Dissolution Studies of the VCM Systems

The in-vitro dissolution characteristics were determined using a method specifically designed for VCM systems [17]. The set-up involves small commercial glasses as dissolution vessels, which comprise the sample and 30 mL of dissolution medium. The glasses are sealed with Parafilm^®^ and placed into an incubator shaker operated at 37 ± 0.5 °C and 130 rpm. To avoid direct contact of the dissolution medium with the drug-loaded core, the curved surface of the VCM samples was covered with acrylate glue (Loctite^®^ 4011, Henkel, Germany), which is impermeable to steroids [23,27] and the aqueous medium [28]. Acetate buffer (pH 4.5) supplemented with 1 wt.% SLS served as dissolution medium. Every 24 ± 0.5 h (or multiples thereof) samples of 1 mL were withdrawn and analyzed for their P4 content via UPLC (Section 2.2.8). The dissolution medium was removed and replaced by fresh pre-heated medium. Taking into account the P4 solubility in the dissolution medium (i.e., 2.5 ± 0.1 mg/mL), the maximum P4 concentration that provides sink conditions is 250 µg/mL. The highest concentration detected in the samples during the drug release studies was 75 µg/mL. All tests were performed for 28 days and every formulation was investigated in triplicate.

#### 2.2.8. UPLC Method

The concentration of P4 was quantified using a reversed-phase UPLC method and a Waters Acquity^®^ H class system. The stationary phase was a Merck Purospher^®^ STAR RP-18 end capped HIBAR^®^ column (2 µm particle size, 100 × 2.1 mm). The column temperature was 50 °C. The mobile phase consisted of ethanol/water 11/9 *v*/*v* and the flow rate was 0.25 mL/min. The injection volume was 4 µL and the total run time was 5 min. P4 was detected at a wavelength of 244 nm. A linear calibration plot was obtained over the concentration range of 0.2–300 µg/mL (R^2^ = 0.9999).

#### 2.2.9. Statistical Analysis

For all methods, triple-fold determinations were carried out. The results are presented as the mean values ± standard deviation (SD). To evaluate statistical significance, Student’s t-tests were performed with a level of *p* < 0.05 being considered as significant (two-sample, unpaired, unequal variance, two-tailed).

## 3. Results

### 3.1. Thermal Analysis of the Pure Components

**Figure 1:**
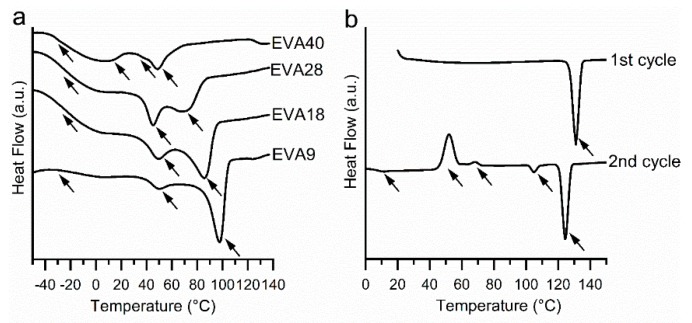
Differential scanning calorimetry (DSC) thermograms (total heat flow, exo up) of (**a**) Ethylene-vinyl acetate (EVA) polymers recorded during the first heating cycle and (**b**) Progesterone (P4) recorded during the first and second heating cycles. The arrows indicate thermal events.

The thermograms of the EVA polymers recorded during the first heating cycle (total signal) are shown in Figure 1a. EVA9 showed two endothermic peaks with an onset at 40 and 88 °C associated with the melting of two crystalline phases. Here, the higher melting phase was more pronounced. With increasing VA content, the onset of the second peak was shifted towards lower temperatures and the lower melting phase became more pronounced. The melting onsets of EVA18 and EVA28 were 39 and 73 °C and 38 and 59 °C, respectively. For EVA40, two major melting events were observed at 34 and 42 °C. In addition, a broad endothermic event was noticeable below 20 °C. The total enthalpy of fusion of the endothermic peaks linearly decreased with increasing VA content (R^2^ > 0.99) due to the decreased polymer crystallinity; i.e., 34, 27, 19 and 6% for EVA9, EVA18, EVA28, and EVA40. These findings are in accordance with studies previously described in the literature [12,13]. The Tg was between –25 to –27 °C, independent of the VA content (determined from the reversing heat flow signal, data not shown). The related specific heat capacity increased with increasing VA content (from 0.084 J/(g*K) for EVA9 to 0.218 J/(g*K) for EVA40). 

Figure 1b shows the thermograms of P4 (total heat flow signal). During the first heating cycle, P4 showed one endothermic event with an onset at 128 °C corresponding to the melting of the thermodynamically stable form I [29,30]. During cooling, no re-crystallization was observed (data not shown). In the second heating cycle, a Tg was observed at 10 °C (determined from the reversing heat flow signal, data not shown). Further heating resulted in P4 cold crystallization at 46 and 65 °C and finally, melting of the metastable forms III (105 °C) and form II (122 °C) [21]. Till date, only form I and II have been fully characterized [21,31,32]. They are both listed in the P4 monograph of the European and United States Pharmacopoeia [32].

### 3.2. P4 solubility in EVA Polymers

**Figure 2:**
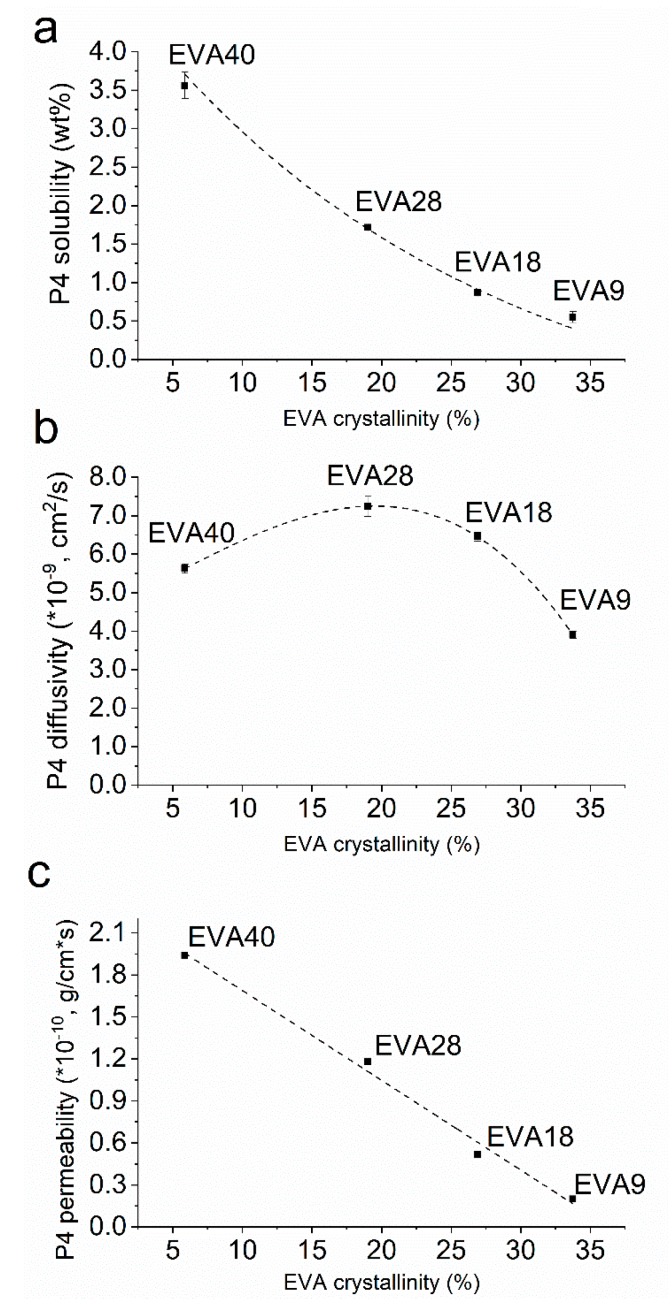
Progesterone (P4) solubility (**a**), diffusivity (**b**), and permeability (**c**) in ethylene-vinyl acetate (EVA) polymers at 37 °C as a function of the polymers’ crystallinity.

**Figure 3:**
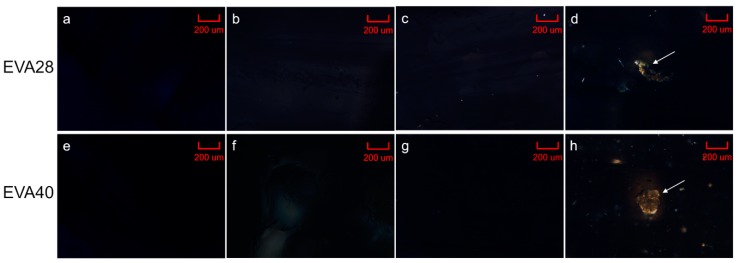
Hot-stage polarized light microscopy images recorded at 90 °C; (**a**) ethylene-vinyl acetate 28 (EVA28), (**b**) EVA28 + 4 wt.% progesterone (P4), (**c**) EVA28 + 5 wt.% P4, (**d**) EVA28 + 6 wt.% P4, (**e**) EVA40, (**f**) EVA40 + 5 wt.% P4, (**g**) EVA40 + 6 wt.% P4 and (**h**) EVA40 + 7 wt.% P4. Arrows indicate the presence of crystals associated with P4. Scale bar: 200 µm.

The equilibrium solubility of P4 in EVA significantly increased with decreasing polymer crystallinity (*p* < 0.05) in a non-linear manner (Figure 2a). Additionally, the effect of thermal treatment on the solubility was determined for EVA28 and EVA40 (i.e., core polymers). EVA28 samples comprising 4 and 5 wt.% P4 did not show drug crystals (Figure 3b,c) and were comparable with pure EVA28 (Figure 3a). By increasing the P4 drug concentration to 6 wt.%, the presence of drug crystals could be confirmed (Figure 3d). EVA40 comprising 5 and 6 wt.% P4 did not reveal any drug crystals either (Figure 3f,g); the samples were also similar to the pure EVA40 polymer (Figure 3e). At 7 wt.% P4 loading, however, drug crystals were detected (Figure 3h). Thus, the P4 solubility after thermal treatment is 5 and 6% in EVA28 and EVA40, respectively.

### 3.3. P4 Diffusivity and Permeability in EVA Polymers

The diffusivity of P4 was a function of the polymers’ crystallinity (Figure 2b). For a VA content between 9 and 28%, the diffusivity significantly increased with decreasing crystallinity (*p* < 0.05). However, for the EVA with the lowest crystallinity, i.e., EVA40, the P4 diffusivity significantly dropped (*p* < 0.05) reaching values that were even below the diffusivity in EVA18. The P4 permeability calculated as the product of the equilibrium solubility and diffusivity increased with decreasing EVA crystallinity in a linear manner (R^2^ > 0.99; Figure 2c). This shows that for a low polymer crystallinity the lower diffusivity was counterbalanced by the higher solubility.

### 3.4. Rational Formulation Design

Table 1 summarizes the VCM formulations that were expected to yield the target release of 400–800 µg/day. The P4 loading in the core was 9 times the corresponding equilibrium solubility yielding 16 and 32% in EVA28 and EVA40, respectively. For EVA28 as core and EVA9 as membrane polymer (corresponding to the NuvaRing^®^ polymer combination, V1_9_100), the target release can only be reached if the P4 solubility in the core is equal to the solubility after thermal treatment. In other words, the P4 must reach a supersaturated state in the core. Increasing the VA content of the membrane to 18% (V1_18_100), the upper limit of the target release can be reached even when the P4 concentration in the core is equivalent to the equilibrium solubility. Increasing the membrane thickness from 100 to 200 µm (V1_18_200) yields half the release equaling the lower target level. 

Using EVA40 as core and EVA9 as membrane, the same release rates were provided as for the EVA28/EVA9 combination (data not shown). The higher P4 solubility in the core polymer is counterbalanced by a considerably lower partition coefficient (i.e., 0.15 for EVA9/EVA40 compared to 0.32 for EVA9/EVA28). Increasing the VA content of the membrane polymer to 18% (V2_18_100) or 28% (V2_28_200 and V2_28_300) yields the entire range of target release by varying the membrane thickness.

Additionally, the total fraction of the drug that is released over 28 days and the corresponding remaining P4 concentration in the core was calculated. Once the P4 concentration drops below its solubility, the P4 concentration gradient across the membrane decreases. Thereby, the daily P4 release gradually diminishes, finally, reaching a level that might be below the target release. In all proposed formulations, the remaining P4 concentration was well above the P4 solubility, when the concentration gradient across the membrane considered the equilibrium solubility (i.e., 1.7 and 3.6 wt.% for EVA28 and EVA40, respectively). However, with Cs being the solubility after thermal treatment, the remaining P4 concentration in the formulations comprising V1 was close to the solubility after solubility treatment (i.e., 5 wt.%). For V1_18_100 even the entire drug is released within 21 days. By contrast, for the formulations containing V2, the remaining P4 concentration was clearly above the solubility after thermal treatment (i.e., 6 wt.%).

### 3.5. Preparation of the VCM systems

**Figure 4:**
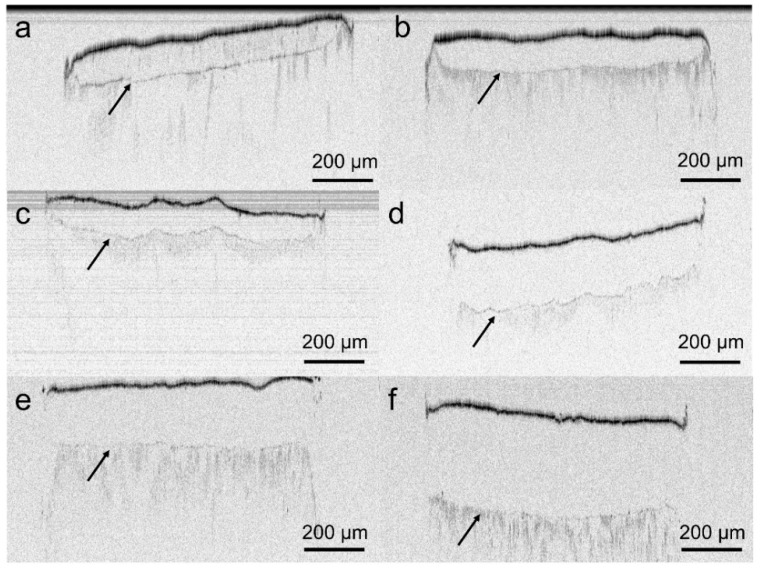
Optical coherence tomography (OCT) images of the formulations; (**a**) V1_9_100, (**b**) V2_18_100, (**c**) V1_18_100, (**d**) V1_18_200, (**e**) V2_28_200, and (**f**) V2_28_300. Arrows indicate the core/membrane interface.

VCM systems were prepared via table top extrusion and the VCM technology. All OCT scans revealed a clear core/membrane interface (Figure 4a–f). The membrane thickness was 99.9 ± 5.7, 100.8 ± 12.2, 201.2 ± 16.1, 91.7 ± 4.9, 202.7 ± 15.9 and 307.1 ± 17.7 µm for V1_9_100, V1_18_100, V1_18_200, V2_18_100, V2_28_200 and V2_28_300. Hence, deviations in the membrane thickness – both, from the target value and within one sample - were in most of cases below 10%.

### 3.6. Solid-State Characterization of the VCM Systems

**Figure 5:**
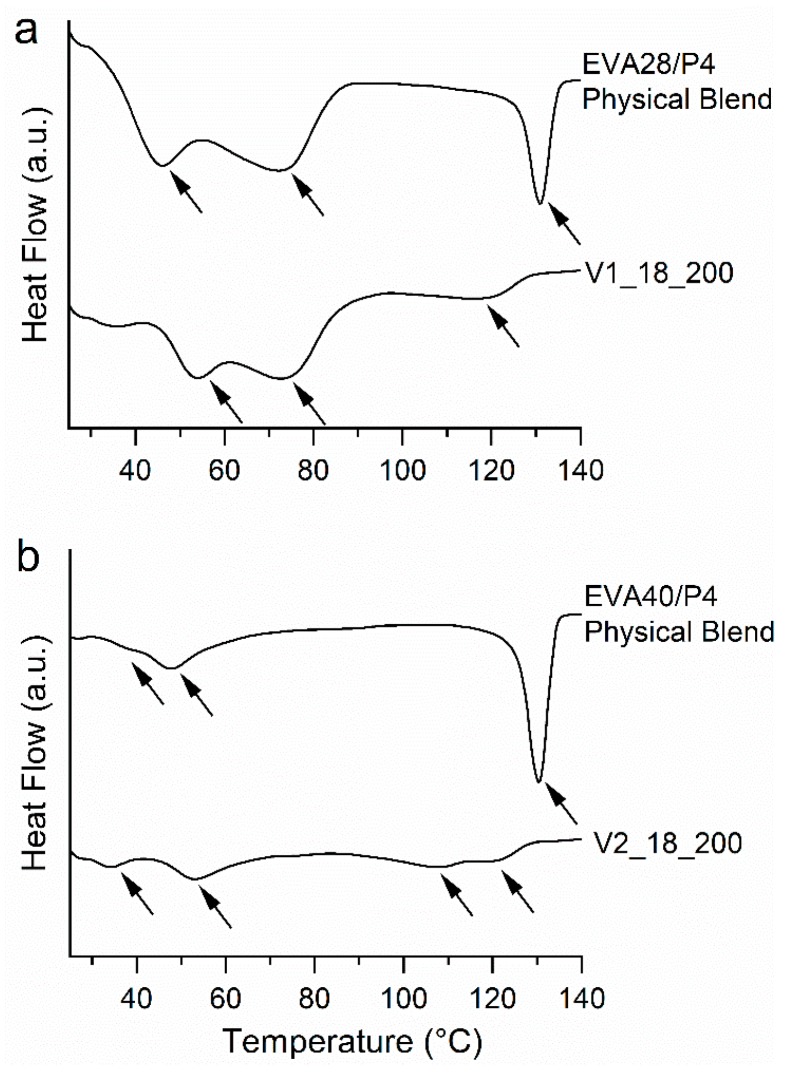
Differential scanning calorimetry (DSC) thermograms of physical blends and vacuum compression molding (VCM) systems: (**a**) EVA28/progesterone (P4) systems, and (**b**) EVA40/P4 systems. Arrows indicate thermal events.

The DSC thermograms of the EVA28/P4 binary blend (Figure 5a) contained 2 endothermic events. Melting of EVA28 and P4 (form I) took place between 36 and 87 °C and at 127 °C (onset), respectively. Similarly, the thermogram of V1_18_200 showed the melting of EVA28 in the temperature range of 47 to 86 °C (Figure 5a). Interestingly, the enthalpy of the EVA28 melting was lower for V1_18_200 compared to the physical blend. These differences can possibly be attributed to surface disorders of EVA caused by embedding crystalline P4 [33]. The surface disorders might cause a reduction of the endothermic signal, especially since EVAs with high VA content tend to crystallize imperfectly [34]. The thermogram of V1_18_200 contained one endothermic peak with an onset at 108 °C that is attributable to the melting of at least one polymorph of P4 (low-melting forms III, IV and V and high melting form II) [21,31,35]. The enthalpy of fusion associated with the P4 melting was rather small compared to the physical blend suggesting that a fraction of P4 was present in its amorphous state. However, HS-PLM images of V1_18_200 (supporting information, Figure S2a,b) that were recorded at temperatures above the melting of EVA (i.e., above 90 °C), revealed crystals assigned to P4. The crystals started melting at temperatures ranging between 110 and 130 °C, which supports the DSC findings as it suggests that they consist of a polymorphic mixture of P4. In addition, the ATR-FTIR spectrum showed peaks at 870 and 864 cm^−1^, associated with P4 form I and II, respectively (supporting information, Figure S1a) [36,37]. The presence of the lower melting forms III-V of P4 could not be confirmed via this technique, as no ATR-FTIR spectra of the lower melting polymorphs are reported in the literature. Comparing the intensities of the peaks at 870 and 864 cm^−1^, the presence of form I was dominating form II.

The DSC thermograms of the EVA40/P4 blend (Figure 5b) revealed solid state properties similar to the EVA28/P4 systems. The physical blend showed the melting of EVA40 (i.e., between 34 and 58 °C) and P4 stable form I (i.e., at 126 °C). Again, the enthalpy of fusion attributed to the EVA40 melting (between 30 and 63 °C) was lower for V2_18_200 due to surface disorders of the polymer. V2_28_200 showed two endothermic events with onsets ranging between 93 and 115 °C associated with the melting of coexisting low- and high-melting P4 polymorphs. The related enthalpy of fusion was rather low compared to the physical blend suggesting that a large fraction of P4 was present in its amorphous state. Similar to the EVA28-based systems, HS-PLM analysis showed the presence of P4 crystals once the EVA polymer was molten above 90 °C (supporting information, Figure S2b,c). Moreover, the ATR-FTIR spectra showed peaks at 870 and 864 cm^−1^ due to the presence of P4 form I and II (supporting information, Figure S1b). From the peak ratios, it appeared that form I was more pronounced than form II. 

Overall, the solid-state characterization revealed that in the VCM systems P4 was present in its amorphous and crystalline state (mixture of several low- and high-melting polymorphs), independent upon the EVA type. The DSC seemed to underestimate the extent of crystalline P4, which was clearly present in a reasonable amount, according to the HS-PLM images and ATR-FTIR spectra. Probably, P4 crystallites were finely dispersed in the EVA matrix, which makes it undetectable via DSC [38].

### 3.7. In-Vitro Drug Dissolution Studies of the VCM Systems

**Figure 6:**
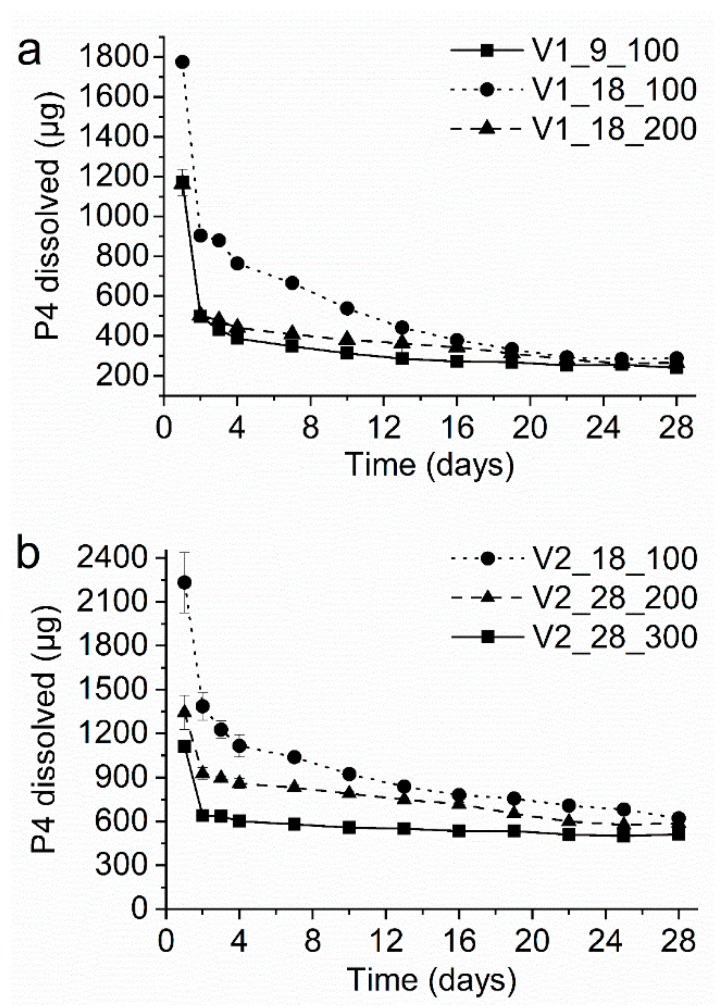
In-vitro progesterone (P4) dissolution profile of the vacuum compression molding (VCM) systems comprising (**a**) EVA28 as core polymer and (**b**) EVA40 as core polymer. The y-axis shows the amount of P4 released per day.

Figure 6a shows the in-vitro dissolution profiles of the VCM systems comprising EVA28 as core polymer (i.e., V1 extrudate). Values for the average daily P4 release (i.e., between day 3 and day 28) and the total P4 fraction released over 28 days are summarized in Table 2 and compared to the predicted values (Table 1). For all tested formulations, a clear burst release (i.e., increased P4 release on day 1) was observed, which is well-known for reservoir systems [39]. For the EVA28/EVA9 core/membrane combination with a membrane thickness of 100 µm (i.e., V1_9_100, based on the NuvaRing^®^ formulation), a slightly declining P4 release profile was obtained. The drug release on day 28 was 56% of the drug release on day 3. The average daily P4 release was 290 µg, which is clearly below a target of 400 µg/day. The average daily release was best predicted when the equilibrium P4 solubility in EVA28 was set as concentration gradient across the membrane instead of the P4 solubility after thermal treatment. This suggests that a pronounced supersaturated state was absent in the core and thus, the target release could not be reached. Changing the membrane polymer to EVA18 (i.e., V1_18_100) and thereby, increasing the P4 solubility and diffusivity in the membrane shifted the release profile towards significantly higher values (*p* < 0.05 for all tested time points). However, the P4 release steadily decreased and finally, the drug release on day 28 was only 33% of the drug release on day 3. The average daily P4 release was 441 µg/day, which is within the target range. However, it is worth noting that the average release is not representative of such a declining profile, as the daily P4 release dropped below 400 µg already on day 16. Consequently, the experimental data were not in agreement with the predicted values. Increasing the EVA18 membrane thickness from 100 to 200 µm (i.e., V1_18_200) resulted in lower drug release rates. The drug release rates were significantly lower (*p* < 0.05) than those observed from V1_18_100 until day 19. Thereafter, the daily P4 release was similar due to the declining release profile of V1_18_100. In contrast to V1_18_100 a more constant release profile was obtained, with the release on day 28 being 55% the value of day 3. The average daily P4 release, i.e., 343 µg/day, was closer to the lower target and well-predicted by the calculations considering the P4 equilibrium solubility in the core.

Overall, the results of the systems containing EVA28 as core polymer showed that it is challenging to reach the target release with this polymer, especially the upper limit. Mainly, this was due to the comparatively low concentration gradient across the membrane, which was best described by the equilibrium P4 solubility in EVA28 and not by the solubility after thermal treatment. Therefore, the core polymer was replaced by EVA40 associated with a higher P4 solubility and a higher P4 concentration gradient across the membrane. Again, all formulations showed a pronounced burst effect (Figure 6b) and the average daily P4 release was well-predicted using the equilibrium P4 solubility in EVA40 for all tested formulations (Table 1). Therefore, a pronounced supersaturated state had not been formed in EVA40, either. Applying an EVA18 membrane of 100 µm thickness (i.e., V2_18_100) yielded a P4 release profile that slightly declined with time (the release on day 28 was 51% of the drug release on day 3). The average daily P4 release was 823 µg/day, which is slightly above the upper target of 800 µg/day. Replacing EVA18 by EVA28 as membrane material and increasing the membrane thickness to 200 µm (i.e., V2_28_200) yielded a significantly lower release profile (*p* < 0.05). The obtained release profile was more stable, as the drug release on day 28 was 65% of the drug release on day 3. The average daily P4 release was 710 µg/day, which is within the target range and close to the upper limit. As anticipated from theoretical predictions, increasing the membrane thickness from 200 to 300 µm (i.e., V2_28_300) significantly decreased the average daily release by about one third yielding 544 µg/day (*p* < 0.05 for all tested time points). Additionally, the release profiles were even more stable with the release on day 28 being 79% of the release on day 3. These results show that the systems comprising EVA40 in the core were capable of providing the target P4 release rates. Both, the upper and the lower were achieved by proper selection of the membrane type in combination with its thickness. Compared to the systems comprising EVA28 as core polymer, the P4 release profiles were more stable over 28 days.

Summarizing, the most promising VCM formulations to reach the release targets of 400 and 800 µg/day were V1_18_200 and V2_18_100, respectively.

## 4. Discussion

In this work, we rationally designed, manufactured and finally, characterized small-scale reservoir systems comprising P4 with the aim to achieve high drug release rates over a period of 4 weeks. For this purpose, the suitability of EVA polymers in terms of reaching the targeted release range was examined through basic physicochemical descriptors. It was found that the P4 equilibrium solubility increased with increasing VA content (Figure 2a), corresponding to a decreased crystallinity of the respective EVA, hence, more amorphous regions were accessible facilitating the dissolution of P4. This behavior has also been shown for other small lipophilic molecules including benzocaine [14], estradiol (E2) [18] ethinyl estradiol and etonogestrel (ENG) [23]. By contrast, the diffusivity of P4 reached a maximum in EVA28, decreased in EVA40 and reached values that were even below those found in EVA18 (Figure 2b). These variations can be attributed to structural alterations of EVA as a function of the VA content [40]. Although the total amorphous volume of the polymer increases with increasing VA content, the amorphous regions become denser [41] and thus, might hinder the diffusion of P4. However, this assumption needs further experimental verification.

Based on the results of the pre-studies and established mathematical models, reservoir concepts were formulated. Different core/membrane polymer combinations were used and the drug release rates were adjusted by varying the membrane thickness. Using EVA28 as core and EVA9 as membrane polymer, the target release was expected to be achieved only if P4 forms a supersaturated solution in EVA28 during HME and/or VCM processing, independent upon the membrane thickness. This seemed very likely, as the solubility after thermal treatment was increased compared to the equilibrium solubility. Additionally, it can be expected that the shear forces present during HME processing may further increase the amount of P4 dissolved in EVA28 [42]. Increasing the VA content of the membrane polymer to 18% suggested that the target release can be provided even if no supersaturated state is created during sample preparation. Here, the increase in P4 release is due to a combination of increased P4 diffusivity in the membrane polymer and an increased P4 partition coefficient between the membrane and the core. In an attempt to further increase the P4 release, EVA40 was selected as core polymer to elevate the concentration gradient across the membrane. Using EVA9 as membrane polymer, however, would not increase the P4 release. The higher concentration gradient is counterbalanced by the lower partition coefficient. In other words, P4 shows a high tendency to remain in the core. Instead, applying EVA18 and EVA28 as membrane materials would considerably increase the P4 release. Using EVA18, the increase is again attributed to the elevated P4 diffusivity in the membrane combined with an increased partition coefficient. By contrast, using EVA28, the higher drug release is only due to the higher partition coefficient, as the diffusivity in EVA18 and EVA28 are very close.

Promising formulations were manufactured and thoroughly characterized. To this end, a small-scale screening procedure that was previously developed by our group and shown to predict the average release from reservoir type IVRs was used [17,18]. Since we have learned in these previous studies that the shear forces in the extruder and/or the cooling process of the extrudates influences the existence of a supersaturated state of the dissolved API in polymer, these conditions were considered and the core was prepared from hot melt extruded pellets. The DSC thermograms generated from the VCM systems showed a much lower enthalpy of fusion attributed to the melting of P4 compared to the physical blends, for both core polymers. Since the P4 loading was nine times the equilibrium solubility, a pronounced supersaturated P4 solution could be assumed. By contrast, the HSM images clearly showed the presence of crystalline P4 suggesting that P4 was finely dispersed in the EVA matrix, which makes it melt within a broad range thus leading to a diffuse melting endotherm in DSC [38].

The absence of a pronounced supersaturated state of drug was further supported by the results of the in-vitro dissolution studies. In the present study, an in-vitro dissolution method specifically designed for VCM systems was applied to ensure sink conditions. Still, it needs to be taken into account that the vaginal fluid is more complex which might slightly impact dissolution characteristics. In all cases, the measured drug release was rather proportional to the equilibrium P4 solubility in the core polymer than to the solubility after thermal treatment. This was contradictory to the results reported for EVA-based systems comprising E2 [18] and ENG [42]. For E2 only low-loaded formulations (i.e., 0.25–1.0%) were considered equaling to a maximum of four times the equilibrium solubility. Although a supersaturated P4 solution in EVA might have been formed during extrusion, the large number of P4 crystals, caused by the high drug loadings (i.e., 16 and 32% for EVA28 and EVA40), likely acted as seed crystals during later cooling and/or storage. Thereby, P4 was prone to re-crystallization and finally, the equilibrium state was reached. For ENG, however, it has been reported that a certain degree of supersaturation (i.e., two times the equilibrium solubility) could be maintained for seven weeks, also in cases where the drug loading was 57-fold the equilibrium solubility (equaling to 20% loading) [42]. The reason for that could be either a higher Tg of ENG or simply a better miscibility with EVA. This shows that whether a supersaturated state is generated in solid state, and more even importantly, maintained is not only a function of the drug loading but also of the drug type. On the one hand, the absence of a supersaturated solution limits the drug release rates that can be achieved. This can become a critical issue especially in cases where high drug release rates are required. On the other hand, the absence of a supersaturated solution is beneficial in terms of product stability. As the system is in its equilibrium state, re-crystallization phenomenon that are likely to occur during storage of supersaturated systems [42] and change their key properties (e.g., in-vitro dissolution characteristics) are absent. Moreover, it is worth mentioning that it is much more difficult to maintain constant drug release from supersaturated systems compared to systems that are in equilibrium. In supersaturated systems, the drug is released and the supersaturated state approaches the equilibrium state. Thereafter, when more drug is released, the drug concentration drops below the equilibrium solubility and drug crystals start dissolving to keep the equilibrium between drug dissolved in the polymer and drug crystals. It is very unlikely that the supersaturated state, which is thermodynamically unstable, is maintained via drug crystal dissolution. As a consequence, the concentration gradient across the membrane – at least initially – drops, which yields declining release profiles.

The absence of a supersaturated state of drug made it challenging to reach the target release – especially the upper limit – from systems comprising EVA28 as core polymer. Although the core/membrane combination of EVA28/EVA9 with a membrane thickness of 100 µm provided rather stable P4 release profiles, the average P4 release was clearly below the target. Replacing EVA9 by EVA18, the P4 release increased as predicted from theoretical considerations. However, the release profiles gradually declined and therefore, the average daily release was 34% below the anticipated value. The calculations assume that the drug concentration in the core is constant, as every dissolved drug molecule that diffuses through the membrane is immediately replaced by dissolving drug crystals. In other words, drug crystal dissolution in the core polymer is not considered the rate-limiting step. However, calculating the P4 concentration in the core from experimental dissolution data and Equation (2), we found that the P4 concentration dropped below the equilibrium solubility already after one week. Thereafter, it steadily decreased finally being only 40% of the equilibrium solubility on day 28. One might assume that there were too few crystals left to be dissolved in the core and that increasing the drug loading would provide a more stable drug release. However, the drug loading was already nine times the equilibrium solubility, which implies that nearly 89% of the incorporated drug were crystalline. In total, 31% of P4 were released, which means that still, the majority of drug was present in its crystalline state. This leads to the conclusion that there was sufficient crystalline drug available to form a reservoir. However, the P4 crystal dissolution kinetics in EVA28 were too slow to counterbalance the comparatively high amounts of P4 released within short time frames. When the membrane thickness was increased from 100 to 200 µm, lower amounts of P4 were released per time unit. This provided more time for P4 crystal dissolution in the core polymer and thus, more constant P4 release rates were achieved.

EVA40 was considered as second core polymer with the aim to increase the P4 release rates. Importantly, EVA40 is likely to provide suitable mechanical characteristics for an IVR [43,44]. As expected, the average daily P4 release was within the target for every tested formulation. With increasing membrane thickness, more stable P4 release profiles were obtained independent upon the membrane polymer type. Increasing the membrane thickness lowers the amount of P4 released per time unit and consequently, provides more time for P4 crystal dissolution in the core polymer Compared to the EVA28 systems, more constant P4 release profiles were obtained. The reason for that may be the different crystal dissolution kinetics of P4 in EVA28 and EVA40. We found that both, EVA28 and EVA40 cores comprised several P4 polymorphs, which could not be quantified via the established methods. Nevertheless, it is likely that the ratio of the individual polymorphs differed in EVA28 and EVA40. Assuming that the P4 crystal morphology and size can differ in different polymers, one can conclude that the P4 crystal dissolution kinetics might vary in EVA28 and EVA40.

Comparing experimental dissolution data to the predicted values, we found that the calculations slightly underpredict the release from systems with low partition coefficients and low membrane thicknesses, i.e., V1_9_100 and V2_18_100. This was attributed to the elevated release at early times that has not been considered in the calculations. The burst (i.e., release on day 1) equaled the amount of P4 that can be dissolved in the membrane at equilibrium plus the average daily P4 release. Hence, the P4 distribution between the core and the membrane was in equilibrium when the samples were subjected to the in-vitro dissolution studies, which occurs rapidly for low membrane thicknesses [45]. Thereafter, the P4 gradually dropped and after 4 days rather constant release profiles were observed. The constant release was attributed to comparatively low amounts of P4 were released per unit time due to the low partition coefficient. On the contrary, the calculations overpredicted the P4 release from systems with high partition coefficients and (comparatively) low membrane thicknesses; i.e., V1_18_100 and V2_28_200. The high amounts of P4 that were initially released yielded a gradually declining P4 concentration in the core and consequently, a steadily declining P4 release profile. For systems having both, a high partition coefficient and a high membrane thickness, i.e., V1_18_200 and V2_28_300, the calculations predicted the experimental results the best. However, it is worth noting that the P4 distribution between the core and the membrane was not in equilibrium when subjected to in-vitro dissolution testing. This was due to the increased membrane thicknesses, which required more time to load the membrane with the drug. Once this equilibrium is reached, it can be expected that the experimental data exceeds the predicted data similar to the systems with a low partition coefficient and a low membrane thickness. Finally, we elucidated critical factors that determine the in-vitro P4 release of the tested reservoir systems. In general, the amount of drug released is a function of (i) the drug partition coefficient between the membrane and the core, (ii) the drug diffusivity in the membrane, (iii) the drug concentration across the membrane (determined by the drug solubility in the core), and (iv) the membrane thickness. For EVA-based reservoir type systems, the amount of P4 that could be released was limited by the P4 solubility in the core polymer. For one core polymer, the P4 release rates were increased by a high P4 partition coefficient and/or a low membrane thickness. In cases where very high P4 amounts were initially released, the crystal dissolution of P4 in the core polymer became the rate-limiting step and thus, the most critical factor. For these formulations, constant P4 release could be maintained only over short time periods.

## 5. Conclusions

The current study demonstrated that high P4 release rates can be achieved from reservoir systems that use EVAs as release controlling polymers. Importantly, to reach the higher release target, an EVA with a VA content of 40% is required in the core. We found that it is challenging to achieve constant drug release over prolonged duration compared to systems with low release rates. This was especially true in cases where high amounts of P4 were released within comparatively short time frames (due to a high P4 partition coefficient between the membrane and core polymer in combination with a low membrane thickness). Here, drug crystal dissolution in the core could not counterbalance drug diffusion through the membrane. The P4 concentration gradient across the membrane gradually decreased and P4 crystal dissolution in the core polymer became the rate-limiting step. As a consequence, the P4 release declined and eventually, dropped below the target. A more constant P4 release was achieved for lower partition coefficients and higher membrane thicknesses, with the latter one having a greater effect. Overall, the design of reservoir systems that provide high drug release rates is more complex compared to the well-known systems delivering low amounts of drug.

## Figures and Tables

**Table 1:** Vacuum compression molding formulations (VCM systems) expected to approach the target progesterone (P4) release rates. All values are calculated based on the results of the pre-studies and Equation (2). Release rates approaching the target of 400–800 µg/day are highlighted in bold.

Abbreviation	Core		Membrane		Cs Is Equilibrium Solubility			Cs is Solubility after Thermal Treatment		
	Polymer Type	P4 Loading (wt.%)	Polymer Type	Thickness (µm)	P4 Release Rate (µg/day)	Fraction Released over 28 Days (%)	Remaining P4 Concentration in the Core after 28 Days (wt.%)	P4 release Rate (µg/day)	Fraction Released over 28 Days (%)	Remaining P4 Concentration in the Core after 28 Days (wt.%)
V1_9_100	EVA28	16	EVA9	100	273	17.0	13.3	**796**	49.5	8.1
V1_18_100	EVA28	16	EVA18	100	**704**	43.8	9.0	2054	>100	n.a.
V1_18_200	EVA28	16	EVA18	200	**352**	21.9	12.5	1027	63.9	5.8
V2_18_100	EVA40	32	EVA18	100	**704**	20.8	25.3	1185	35.0	20.1
V2_28_200	EVA40	32	EVA28	200	**802**	23.7	24.4	1350	39.9	19.2
V2_28_300	EVA40	32	EVA28	300	**534**	15.8	26.9	**899**	26.6	23.5

**Table 2:** In-vitro progesterone (P4) dissolution data generated from the VCM systems. * The average daily release was calculated between day 3 and day 28. ** Predicted values using the equilibrium P4 solubility in the core polymer and the measured membrane thickness in Equation (2) (Section 3.4).

Abbreviation	Average Experimental Daily P4 Release (µg/day) * (S.D.)	Predicted Daily P4 Release (µg/Day) **	Deviation Experimental from Predicted (%)	P4 Fraction Released over 28 Days (% of Loading)
V1_9_100	289.8 (47.5)	273.2	+6.1	20.6
V1_18_100	440.8 (163.7)	698.4	-36.9	31.4
V1_18_200	343.1 (56.5)	349.9	-2.0	23.5
V2_18_100	822.6 (159.9)	767.7	+7.2	26.4
V2_28_200	710.2 (95.0)	790.9	-10.2	22.2
V2_28_300	543.7 (32.0)	522.0	+4.2	17.1

## Supplementary Materials

The following are available online at https://www.mdpi.com/1999-4923/12/2/103/s1, Figure S1: Attenuated total reflectance Fourier transform infrared spectroscopy (ATR-FTIR) spectra of (a) EVA28/progesterone (P4) systems and (b) EVA40/P4 systems. The solid arrows indicate the peaks corresponding to form I of P4 (870 cm^−1^). The dotted arrows indicate the peaks corresponding to form II of P4 (864 cm^−1^)., Figure S2: Hot-stage polarized light microscopy (HS-PLM) images of (a) V1_18_200 recorded at 115 °C; (b) V1_18_200 recorded at 130 °C, (c) V2_18_200 recorded at 115 °C and (d) V2_18_200 recorded at 130 °C. The white arrows indicate P4 crystals.

## References

[1] Jones D. (2004). Pharmaceutical Applications of Polymers for Drug Delivery (Rapra Review Reports).

[2] Yang W.W., Pierstorff E. (2012). Reservoir-based polymer drug delivery systems. J. Lab. Autom..

[3] Fan L., Singh S.K., Cantow H.-J., Harwood H.J., Kennedy J.P., Ledwith A., Meissner J., Okamura S., Henrici-Olive G., Olive S. (1989). Controlled Release A Quantitative Treatment.

[4] Siepmann J., Siegel R.A., Siepmann F., Siepmann J., Siegel R.A., Rathbone M.J. (2012). Diffusion Controlled Drug Delivery Systems. Fundamentals and Applications of Controlled Release Drug Delivery.

[5] Nel A., Smythe S., Young K., Malcolm K., McCoy C., Rosenberg Z., Romano J. (2009). Safety and pharmacokinetics of dapivirine delivery from matrix and reservoir intravaginal rings to HIV-negative women. J. Acquir. Immune Defic. Syndr..

[6] Clark J.T., Clark M.R., Shelke N.B., Johnson T.J., Smith E.M., Andreasen A.K., Nebeker J.S., Fabian J., Friend D.R., Kiser P.F. (2014). Engineering a segmented dual-reservoir polyurethane intravaginal ring for simultaneous prevention of HIV transmission and unwanted pregnancy. PLoS ONE.

[7] Brache V., Payán L.J., Faundes A. (2013). Current status of contraceptive vaginal rings. Contraception.

[8] Johnson T.J., Clark M.R., Albright T.H., Nebeker J.S., Tuitupou A.L., Clark J.T., Fabian J., McCabe R.T., Chandra N., Doncel G.F. (2012). A 90-day tenofovir reservoir intravaginal ring for mucosal HIV prophylaxis. Antimicrob. Agents Chemother..

[9] Holmgren P.Å., Lindskog M., von Schoultz B. (1989). Vaginal rings for continuous low-dose release of oestradiol in the treatment of urogenital atrophy. Maturitas.

[10] Croxatto H.B. (2000). Progestin implants. Steroids.

[11] Sivin I., Mishell D.R., Alvarez F., Brache V., Elomaa K., Lähteenmäki P., Massai R., Miranda P., Croxatto H., Dean C. (2005). Contraceptive vaginal rings releasing Nestorone^®^ and ethinylestradiol: A 1-year dose-finding trial. Contraception.

[12] Schneider C., Langer R., Loveday D., Hair D. (2017). Applications of ethylene vinyl acetate copolymers (EVA) in drug delivery systems. J. Control. Release.

[13] Almeida A., Possemiers S., Boone M.N., De Beer T., Quinten T., Van Hoorebeke L., Remon J.P., Vervaet C. (2011). Ethylene vinyl acetate as matrix for oral sustained release dosage forms produced via hot-melt extrusion. Eur. J. Pharm. Biopharm..

[14] Chen S.X., Lostritto R.T. (1996). Diffusion of benzocaine in poly(ethylene-vinyl acetate) membranes: Effects of vehicle ethanol concentration and membrane vinyl acetate content. J. Control. Release.

[15] Hamada A.L., Maruo T., Samoto T., Yoshida S., Nash H., Spitz I.M., Johansson E. (2003). Estradiol/progesterone-releasing vaginal rings for hormone replacement therapy in postmenopausal women. Gynecol. Endocrinol..

[16] Matlin S.A., Beleuguer A., Hall P.E. (1992). Progesterone-Releasing Vaginal Rings for Use in Postpartum Contraception. Contraception.

[17] Eder S., Beretta M., Witschnigg A., Koutsamanis I., Eggenreich K., Khinast J.G., Koscher G., Paudel A., Nickisch K., Friedrich M. (2017). Establishment of a Molding Procedure to Facilitate Formulation Development for Co-extrudates. AAPS PharmSciTech.

[18] Koutsamanis I., Eder S., Beretta M., Witschnigg A., Paudel A., Nickisch K., Friedrich M., Eggenreich K., Roblegg E. (2019). Formulation and processability screening for the rational design of ethylene-vinyl acetate based intra-vaginal rings. Int. J. Pharm..

[19] Wunderlich B., Czornyj G. (1977). A Study of Equilibrium Melting of Polyethylene. Macromolecules.

[20] Helbling I.M., Ibarra J.C.D., Luna J.A. (2014). The optimization of an intravaginal ring releasing progesterone using a mathematical model. Pharm. Res..

[21] Lancaster R.W., Karamertzanis P.G., Hulme A.T., Tocher D.A., Lewis T.C., Price S.L. (2007). The Polymorphism of Progesterone: Stabilization of a ‘Disappearing’ Polymorph by Co-Crystallization. J. Pharm. Sci..

[22] Siepmann J., Lecomte F., Bodmeier R. (1999). Diffusion-controlled drug delivery systems: Calculation of the required composition to achieve desired release profiles. J. Control. Release.

[23] Van Laarhoven J.A.H., Kruft M.A.B., Vromans H. (2002). In vitro release properties of etonogestrel and ethinyl estradiol from a contraceptive vaginal ring. Int. J. Pharm..

[24] Roumen F.J.M.E., Dieben T.O.M. (1999). Clinical acceptability of an ethylene-vinyl-acetate nonmedicated vaginal ring. Contraception.

[25] Krier F., Mantanus J., Sacré P.Y., Chavez P.F., Thiry J., Pestieau A., Rozet E., Ziemons E., Hubert P., Evrard B. (2013). PAT tools for the control of co-extrusion implants manufacturing process. Int. J. Pharm..

[26] Schneider C.A., Rasband W.S., Eliceiri K.W. (2012). NIH Image to ImageJ: 25 years of image analysis. Nat. Methods.

[27] Externbrink A., Eggenreich K., Eder S., Mohr S., Nickisch K., Klein S. (2017). Development and evaluation of accelerated drug release testing methods for a matrix-type intravaginal ring. Eur. J. Pharm. Biopharm..

[28] LOCTITE^®^ 4011TM Technical Data Sheet. https://tdsna.henkel.com/NA/UT/HNAUTTDS.nsf/web/40B3DBCA6D797572882571870000D75F/$File/4011-EN.pdf.

[29] Duclos R., Saiter J.M., Grenet J., Orecchioni A.M. (1991). Polymorphism of progesterone - Influence of the carrier and of the solid dispersion manufacturing processes. A calorimetric and radiocrystallographic study. J. Therm. Anal..

[30] Wang F., Wachter J.A., Antosz F.J., Berglund K.A. (2000). An Investigation of Solvent-Mediated Polymorphic Transformation of Progesterone Using in Situ Raman Spectroscopy. Org. Proc. Res. Dev..

[31] Legendre B., Feutelais Y., Defossemont G. (2003). Importance of heat capacity determination in homogeneous nucleation: Application to progesterone. Thermochim. Acta.

[32] Sarkar A., Ragab D., Rohani S. (2014). Polymorphism of progesterone: A new approach for the formation of form II and the relative stabilities of form i and form II. Cryst. Growth Des..

[33] Wang L., Fang F., Ye C., Feng J. (2004). Solid-State NMR Characterizations on Phase Structures and Molecular Dynamics of Poly(ethylene-co-vinyl acetate). J. Polym. Sci. Part B Polym. Phys..

[34] Shi X.M., Zhang J., Jin J., Chen S.J. (2008). Non-isothermal crystallization and melting of ethylene-vinyl acetate copolymers with different vinyl acetate contents. Express Polym. Lett..

[35] Brandstaetter-Kuhnert M., Kofler A. (1959). Zur mikroskopischen Identitaetspruefung und zur Polymorphie der Sexualhormone. Microchim. Acta.

[36] Mesley R.J. (1966). The infra-red spectra of steroids in the solid state. Spectrochmica Acta.

[37] Araya-Sibaja A.M., Paulino A.S., Rauber G.S., Maduro Campos C.E., Cardoso S.G., Monti G.A., Heredia V., Bianco I., Beltrano D., Cuffini S.L. (2014). Dissolution properties, solid-state transformation and polymorphic crystallization: Progesterone case study. Pharm. Dev. Technol..

[38] Helbling I.M., Ibarra J.C.D., Luna J.A. (2016). The Use of Cellulose Membrane to Eliminate Burst Release from Intravaginal Rings. AAPS J..

[39] Huang X., Brazel C.S. (2001). On the importance and mechanisms of burst release in matrix-controlled drug delivery systems. J. Control. Release.

[40] Maurin M.B., Dittert L.W., Hussain A.A. (1992). Mechanism of diffusion of monosubstituted benzoic acids through ethylene-vinyl acetate copolymers. J. Pharm. Sci..

[41] Dlubek G., Lüpke T., Stejny J., Alam M.A., Arnold M. (2000). Local free volume in ethylene-vinyl acetate copolymers: A positron lifetime study. Macromolecules.

[42] Van Laarhoven J.A.H., Kruft M.A.B., Vromans H. (2002). Effect of supersaturation and crystallization phenomena on the release properties of a controlled release device based on EVA copolymer. J. Control. Release.

[43] Boyd P., Major I., Wang W., McConville C. (2014). Development of disulfiram-loaded vaginal rings for the localised treatment of cervical cancer. Eur. J. Pharm. Biopharm..

[44] Singer R., Mawson P., Derby N., Rodriguez A., Kizima L., Menon R., Goldman D., Kenney J., Aravantinou M., Seidor S. (2012). An Intravaginal Ring That Releases the NNRTI MIV-150 Reduces SHIV Transmission in Macaques. Sci. Transl. Med..

[45] Siepmann J., Siepmann F. (2012). Modeling of diffusion-controlled drug delivery. J. Control. Release.

