# Supplementary material for: Controlled-Release from High-Loaded Reservoir-Type Systems—A Case Study of Ethylene-Vinyl Acetate and Progesterone
Source: Pharmaceutics. 2020 Jan 28;12(2):103. doi: 10.3390/pharmaceutics12020103 (PMC7076478; doi:10.3390/pharmaceutics12020103)
Supplement: Supplementary file 1 [file pharmaceutics-12-00103-s001.pdf]

pharmaceutics

Supplementary Materials: Controlled-Release from High-Loaded Reservoir-Type Systems – A Case Study of Ethylene-Vinyl Acetate and Progesterone

Ioannis Koutsamanis, Amrit Paudel, Klaus Nickisch, Karin Eggenreich, Eva Roblegg and Simone Eder

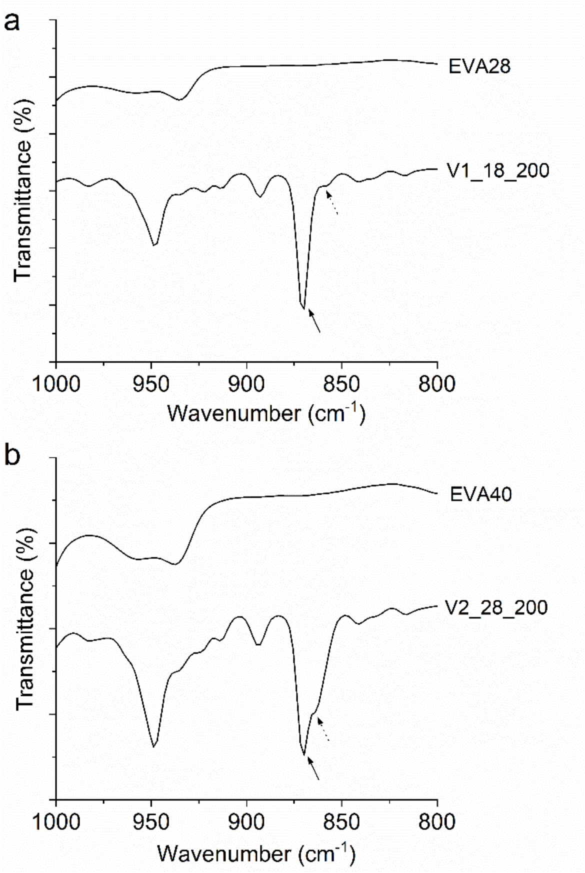

Figure S1. Attenuated total reflectance Fourier transform infrared spectroscopy (FTIR) spectra of (**a**) EVA28/progesterone (P4) systems and (**b**) EVA40/P4 systems. The solid arrows indicate the peaks corresponding to form I of P4 (870 cm^{-1}). The dotted arrows indicate the peaks corresponding to form II of P4 (864 cm^{-1}).

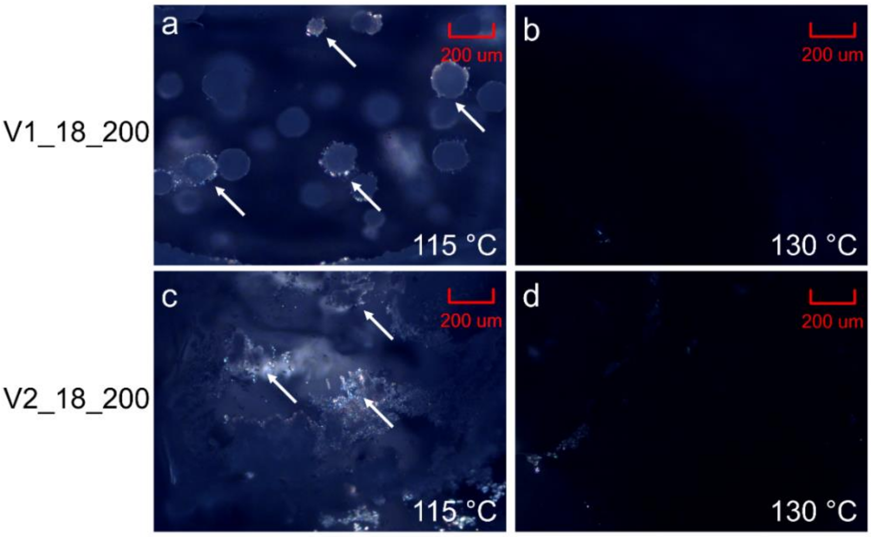

Figure S2. Hot-stage polarized light microscopy (HS-PLM) images of (**a**) V1_18_200 recorded at 115 °C; (**b**) V1_18_200 recorded at 130 °C, (**c**) V2_18_200 recorded at 115 °C and (**d**) V2_18_200 recorded at 130 °C. The white arrows indicate progesterone crystals.
